# Inhibition of GLS1 and ASCT2 Synergistically Enhances the Anticancer Effects in Pancreatic Cancer Cells

**DOI:** 10.4014/jmb.2412.12032

**Published:** 2025-04-10

**Authors:** Dong-Hwan Kim, Dong Joon Kim, Seong-Jun Park, Won-Jun Jang, Chul-Ho Jeong

**Affiliations:** 1 College of Pharmacy, Keimyung University, Daegu 42601, Republic of Korea; 2 Department of Microbiology, College of Medicine, Dankook University, Cheonan 31116, Republic of Korea

**Keywords:** Pancreatic cancer, glutamine metabolism, CB-839, V-9302, ATF4

## Abstract

Pancreatic cancer, a leading cause of cancer-related deaths, is characterized by increased dependence on glutamine metabolism. Telaglenastat (CB-839), a glutaminase (GLS) inhibitor targets glutamine metabolism; however, its efficacy as monotherapy is limited owing to metabolic adaptations. In this study, we demonstrated that CB-839 effectively inhibited cell growth in pancreatic cancer cells, but activated the general control nonderepressible 2 (GCN2)-activating transcription factor 4 (ATF4) signaling pathway. ATF4 knockdown reduced glutamine transporter alanine, serine, and cysteine transporter 2 (ASCT2) expression, glutamine uptake, and cell viability under glutamine deprivation-recovery conditions, confirming its protective role in mitigating glutamine-related metabolic stress. Notably, the combination of CB-839 and the ASCT2 inhibitor V-9302 demonstrated a synergistic effect, significantly suppressing pancreatic cancer cell survival. These findings highlight ATF4 and ASCT2 as crucial therapeutic targets and indicate that dual inhibition of GLS and ASCT2 may enhance treatment outcomes for pancreatic cancer.

## Introduction

Pancreatic cancer is challenging to diagnose and is associated with poor prognosis, with a five-year survival rate of < 10%. Conventional strategies, such as gemcitabine and radiotherapy, do not significantly improve survival rates, highlighting the need for novel approaches [1-3]. Numerous cancer cells, including pancreatic cancer cells, are highly dependent on glutamine metabolism to meet their energy requirements, generate essential biosynthetic precursors, and maintain redox homeostasis [4]. Therefore, targeting glutamine metabolism has emerged as a promising strategy in cancer therapy [5]. The conversion of glutamine to glutamate by glutaminase (GLS) is a crucial step in the tricarboxylic acid cycle and supports multiple anabolic processes. Consequently, numerous GLS inhibitors, including 6-diazo-5-oxo-L-norleucine, bis-2-(5-phenylacetamido-1,2,4-thiadiazol-2-yl)ethyl sulfide, and compound 968, were developed to target this pathway [6-8]. However, these initial GLS inhibitors exhibited limited clinical utility owing to their significant cytotoxicity and poor specificity for the tetrameric form of GLS [7].

The development of telaglenastat (CB-839), a novel allosteric inhibitor of GLS, signifies a substantial advancement in targeting glutamine metabolism with enhanced specificity and diminished toxicity. Preclinical studies demonstrated the potential of CB-839 and were assessed in Phase I and Phase II clinical trials in patients with advanced or metastatic solid tumors, including triple-negative breast cancer and renal cell carcinoma (RCC) [9-11]. However, the intrinsic metabolic adaptability of cancer cells poses a significant challenge to the efficacy of CB-839 monotherapy [12]. Cancer cells exhibit significant metabolic plasticity, adapting to nutrient deprivation by activating alternative pathways, thereby limiting the efficacy of metabolic anticancer agents, such as CB-839.

CB-839 administration inhibits GLS activity, thereby inducing amino acid deprivation stress within the cell. This stress activates the general control non-repressible 2 (GCN2) kinase pathway that detects uncharged transfer RNA (tRNA) as an amino acid starvation signal [13, 14]. Upon activation, GCN2 phosphorylates eukaryotic initiation factor 2 alpha (eIF2α) to selectively facilitate the translation of stress-responsive proteins, including activating transcription factor 4 (ATF4), while inhibiting global protein synthesis [14, 15]. ATF4 serves as a primary regulator of the integrated stress response (ISR), modulating the expression of genes involved in amino acid transport, redox balance, and cellular adaptation under stress [15]. Depending on the context and duration of the stress signal, ATF4 can induce both pro-survival and pro-apoptotic pathways. For example, ATF4 induces cell cycle arrest and apoptosis by upregulating pro-apoptotic factors. Additionally, it enhances cell survival by increasing amino acid transporters, such as SLC1A5 (alanine-serine-cysteine transporter 2, ASCT2) to replenish intracellular amino acids [14]. During stress, increased ATF4 levels enhanced ASCT2 expression, enabling cancer cells to enhance glutamine uptake and sustain growth [16, 17]. Therefore, because of the crucial role of ATF4 under glutamine stress conditions, the modulation of ATF4 and ASCT2 may offer a promising therapeutic target for cancer treatment.

In this study, we assessed the potential role of ATF4 in CB-839-induced pancreatic cancer cell resistance and explored strategies to enhance CB-839 efficacy in pancreatic cancer cells. Additionally, we aimed to explore the role of ATF4 under glutamine metabolic stress and reasoned that ATF4 upregulates ASCT2, a glutamine transporter, thereby enhancing the survival of pancreatic cancer cells. Therefore, we suggest that ASCT2 inhibition alongside CB-839 treatment may enhance CB-839 effectiveness by reducing glutamine uptake and overcoming resistance in pancreatic cells.

## Materials and Methods

### Chemicals and Reagents

CB-839 and V-9302 were purchased from MedChemExpress (USA). Dimethyl sulfoxide (DMSO) and crystal violet were purchased from Sigma-Aldrich (USA). Antibodies against GCN2, p-GCN2, eIF2α, p-eIF2α, ATF4, β-actin, ASCT2, caspase 7, p53 upregulated modulator of apoptosis (PUMA), and poly (ADP-ribose) polymerase (PARP), as well as the corresponding secondary antibodies, were obtained from Cell Signaling Technology (USA). The Glutamine/Glutamate-Glo Assay kit was purchased from Promega (USA).

### Cell lines and Cell Culture

Human pancreatic adenocarcinoma BxPC-3 and HPAC cell lines were obtained from the American Type Culture Collection (USA) and were maintained in Roswell Park Memorial Institute (RPMI) 1640 medium supplemented with 10% fetal bovine serum (Atlas Biologicals, USA) and 1% penicillin-streptomycin (HyClone Laboratories, USA). The BxPC-3 and HPAC cell lines were cultured in a humidified incubator at 37°C under 5%CO_2_, following standard laboratory practices.

### Cell Viability Assay

Cell viability was assessed using the 3-(4,5-dimethylthiazol-2-yl)-2,5-diphenyltetrazolium bromide (MTT) assay. The specified cells were seeded in 96-well plates in RPMI 1640 medium and incubated overnight. Subsequently, the cells were treated with the drug and incubated for 48 h in a humidified incubator at 37°C under 5% CO_2_. MTT (20 μl) was added to each well, and the cells were further incubated for 2 h. The medium was removed and 200 μl of DMSO was added to each well. The absorbance was measured at 570 nm using a FLUOstar Omega (BMG Labtech, Germany), with a reference at 630 nm.

### Colony Formation Assay

The BxPC-3 and HPAC cell lines were seeded in 6-well cell culture plates at a density of 1 × 10^4^ and 1 × 10^3^ cells/well, respectively, in RPMI 1640 medium. Throughout the experiment, the plates were maintained in a humidified incubator at 37°C under 5% CO_2_. After a 4-day incubation, the cells were treated with the indicated drug concentrations in glutamine-free medium. Following an additional 5-day incubation, the cells were washed twice with Dulbeccós phosphate-buffered saline (DPBS), fixed with methanol for 3 min, washed once more with DPBS, and stained with 1% crystal violet. Colony densities were calculated using NIH ImageJ software.

### Western Blotting

BxPC-3 and HPAC cells were separately plated in 100-mm cell culture dishes in RPMI 1640 medium and incubated in a humidified incubator at 37°C under 5% CO_2_ for 24 h. Subsequently, the cells were treated with CB-839 or maintained in glutamine-free RPMI 1640 medium for 24–72 h. The cells were then lysed using chilled whole-cell lysis buffer or radioimmunoprecipitation assay (RIPA) buffer supplemented with Halt protease inhibitor cocktail, phenylmethylsulfonyl fluoride (PMSF), and ethylenediaminetetraacetic acid (EDTA). All lysis reagents were obtained from Sigma-Aldrich. Each sample was subjected to ultrasonication for 1.5 s, repeated thrice. Subsequently, the samples were centrifuged (16,000 ×*g*, 20 min, at 4°C). The total protein content of the cell lysate was determined using a bicinchoninic acid (BCA) protein assay kit (Thermo Fisher Scientific, USA). Equal amounts of total protein were loaded into each well, separated by sodium dodecyl sulfate-polyacrylamide gel electrophoresis (SDS-PAGE), and transferred to polyvinylidene fluoride (PVDF) membranes (GE Healthcare, USA). The membranes were blocked with 5% skim milk in Tris-buffered saline with Tween 20 (TBS-T) and incubated with primary antibodies overnight at 4°C. Subsequently, the membranes were washed four times with TBS-T and incubated with species-specific horseradish peroxidase-conjugated secondary antibodies. Protein bands were visualized using SuperSignal West Dura Extended Duration Substrate (Thermo Fisher Scientific) and imaged using a LAS-3000 (Fuji Film, Japan), following the manufacturer's protocol.

### siRNA Transfection and Knockdown

Double-stranded small interfering RNA (siRNA) targeting human ATF4 (GenBank Accession No. NM_001675.4) was synthesized by Bioneer Corporation (Republic of Korea). A negative control siRNA (Cat. No. SN-1003) was also obtained from Bioneer. The specific ATF4 siRNA sequences were as follows:

Sense (forward): 5'-CGU UGC UGU AAC CGA CAA A-3'

Antisense (reverse): 5'-UUU GUC GGU UAC AGC AAC G-3'

BxPC-3 and HPAC cells were seeded in the recommended culture medium at 1.5 × 10^3^ and 1 × 10^3^ cells per well, respectively, in 96-well plates, and at 1 × 10^4^ and 1 × 10^3^ cells per well, respectively, in 6-well plates. The cells were incubated overnight at 37°C in a humidified incubator containing 5% CO_2_. On the following day, the cells were transfected with either ATF4 siRNA or negative control siRNA using Oligofectamine transfection reagent (Invitrogen, USA), according to the manufacturer’s instructions. Knockdown efficiency was determined by measuring the reduction in ATF4 protein levels via Western blot analysis. The functional effect of ATF4 knockdown was subsequently evaluated by examining changes in downstream protein expression and by measuring cell viability using the MTT assay.

### Glutamate Assay

BxPC-3 and HPAC cell lines were seeded in 24-well plates and transfected with ATF4 or negative control siRNAs. Subsequently, the medium was replaced with 2 ml of glutamine-free medium to induce ATF4 expression for 24 h, followed by 2 ml of medium with and without glutamine. After three days, 10 μl of the medium was extracted, diluted with 490 μl of filtered triple-distilled water, and transferred to 96-well white luminescent plates for glutamate detection. Luminescence was measured using the Glutamine/Glutamate Glo Assay (#J8021; Promega), following the prescribed protocol and quantified using Fluostar Omega (BMG Labtech, Germany). The error in glutamate measurements was corrected using an MTT assay at 570 nm, following the prescribed protocol.

### Drug Combination Analysis

The synergistic effects of CB-839 and V-9302 were analyzed using the SynergyFinder web-tool (https://synergyfinder.org/). Dose-response matrices were generated by treating cells with various concentrations of the drugs, both individually and in combination. The observed responses were compared to the expected effects calculated using the Highest Single Agent (HSA) model, which assumes the combination effect equals the maximum effect of either drug alone. All calculations were performed on the SynergyFinder platform.

### Statistical Analysis

Statistical analyses were performed using GraphPad Prism 8 (GraphPad Software Inc., USA). Differences between groups were analyzed using Student's *t*-test, one-way analysis of variance (ANOVA) or two-way ANOVA with statistical significance set at *p* < 0.05. The data are presented as the mean ± standard deviation (SD) from at least three independent experiments, each performed in triplicate.

## Results

### CB-839 Reduces Cell Viability of Pancreatic Cancer Cells

**Fig. 1:**
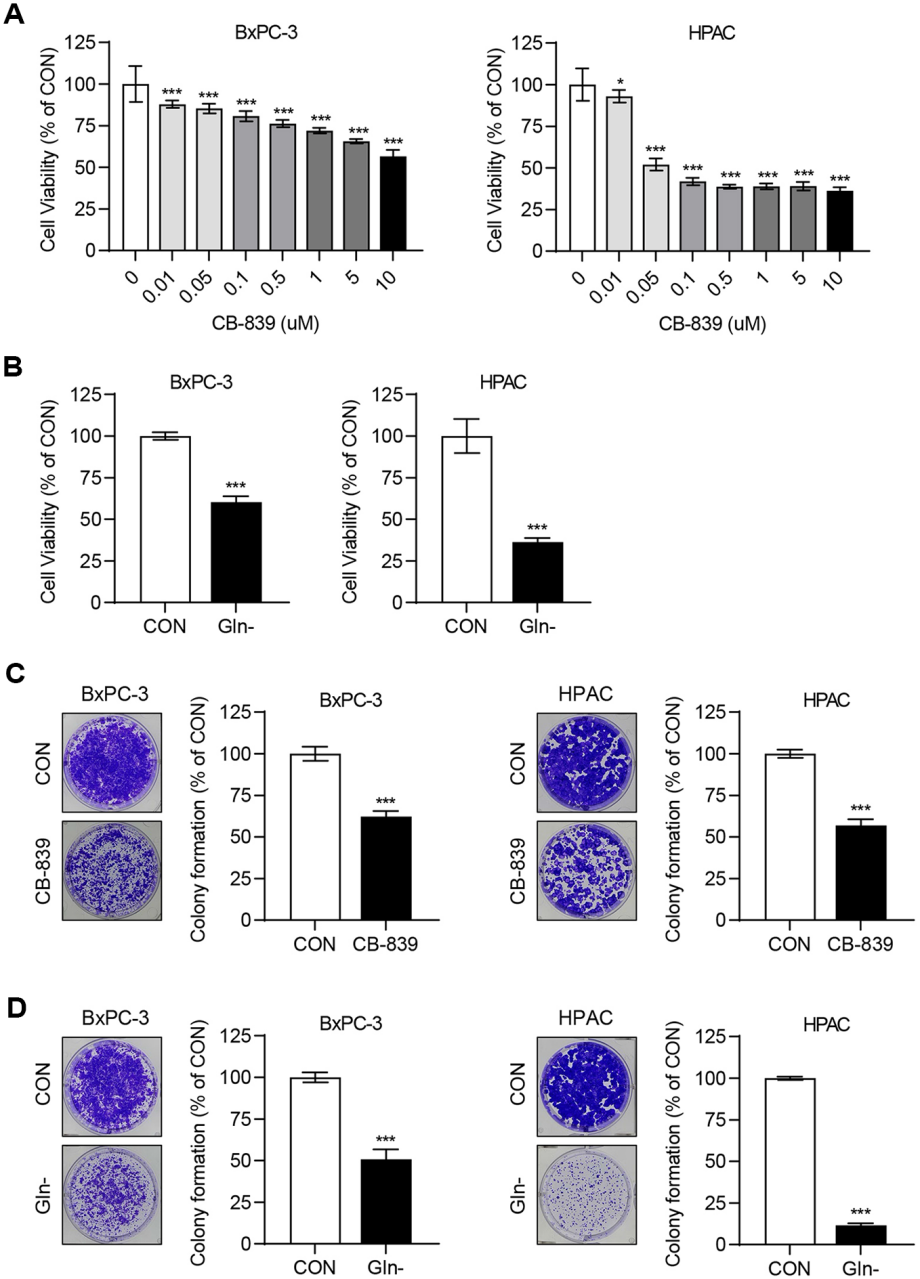
CB-839 treatment or glutamine deprivation suppresses cell viability and colony formation in pancreatic cancer cells. (**A**, **B**) BxPC3 and HPAC cells were treated with various concentrations of CB-839 or cultured under glutamine deprivation conditions for three days. Cell viability was measured using the MTT assay, and statistical analyses were performed using one-way ANOVA and Student’s *t*-test in GraphPad Prism 8. Error bars represent the mean ± SD (*n* = 6). *p < 0.05 and ***p < 0.001 compared with the control (CON) group. (**C**, **D**) BxPC-3 and HPAC cells were seeded in 6- well plates at densities of 1 × 10^4^ and 1 × 10^3^ cells/well, respectively, in 6-well plates and cultured for 4 days. The cells were then treated with 1 μM CB-839 or cultured under glutamine-deprived conditions for 5 days. Colonies were fixed with methanol, stained with crystal violet, and quantified using ImageJ. Statistical analyses were performed using Student’s *t*-test in GraphPad Prism 8. Error bars represent the mean ± SD (*n* = 3). ***p < 0.001 compared with the CON group.

Pancreatic cancer cells are highly dependent on glutamine metabolism for survival and rapid proliferation [18-21]. Therefore, treatment with CB-839 or glutamine deprivation may reduce cell viability. To test this, BxPC-3 and HPAC cell lines were seeded in 96-well plates and treated with CB-839 (0–10 μM) or cultured under glutamine deprivation conditions. After three days, the MTT assay results revealed a dose-dependent reduction in cell viability following CB-839 treatment, with a significant reduction under glutamine deprivation (Fig. 1A and 1B). However, lower concentrations of CB-839 did not completely reduce cell viability, indicating that targeting GLS1 alone may be insufficient. This observation is consistent with the findings of previous studies [12].

### CB-839 Reduces Colony Formation in Pancreatic Cancer Cells

Although the MTT assays confirmed the reduced viability of BxPC-3 and HPAC cells following CB-839 treatment or glutamine deprivation, the effect was incomplete. To further assess this, a colony formation assay was conducted. Cells were seeded in 6-well plates, cultured for four days, and treated with 1 uM CB-839 or subjected to glutamine deprivation for five days. The colonies were fixed with methanol and stained with crystal violet. The results demonstrated that CB-839 inhibited colony formation, although less effectively than that of glutamine deprivation. This indicated that CB-839 alone may be insufficient to fully suppress pancreatic cancer cell survival (Fig. 1C and 1D).

### Enhanced ATF4 Expression Following GLS1 Inhibition Facilitates Cell Survival

**Fig. 2:**
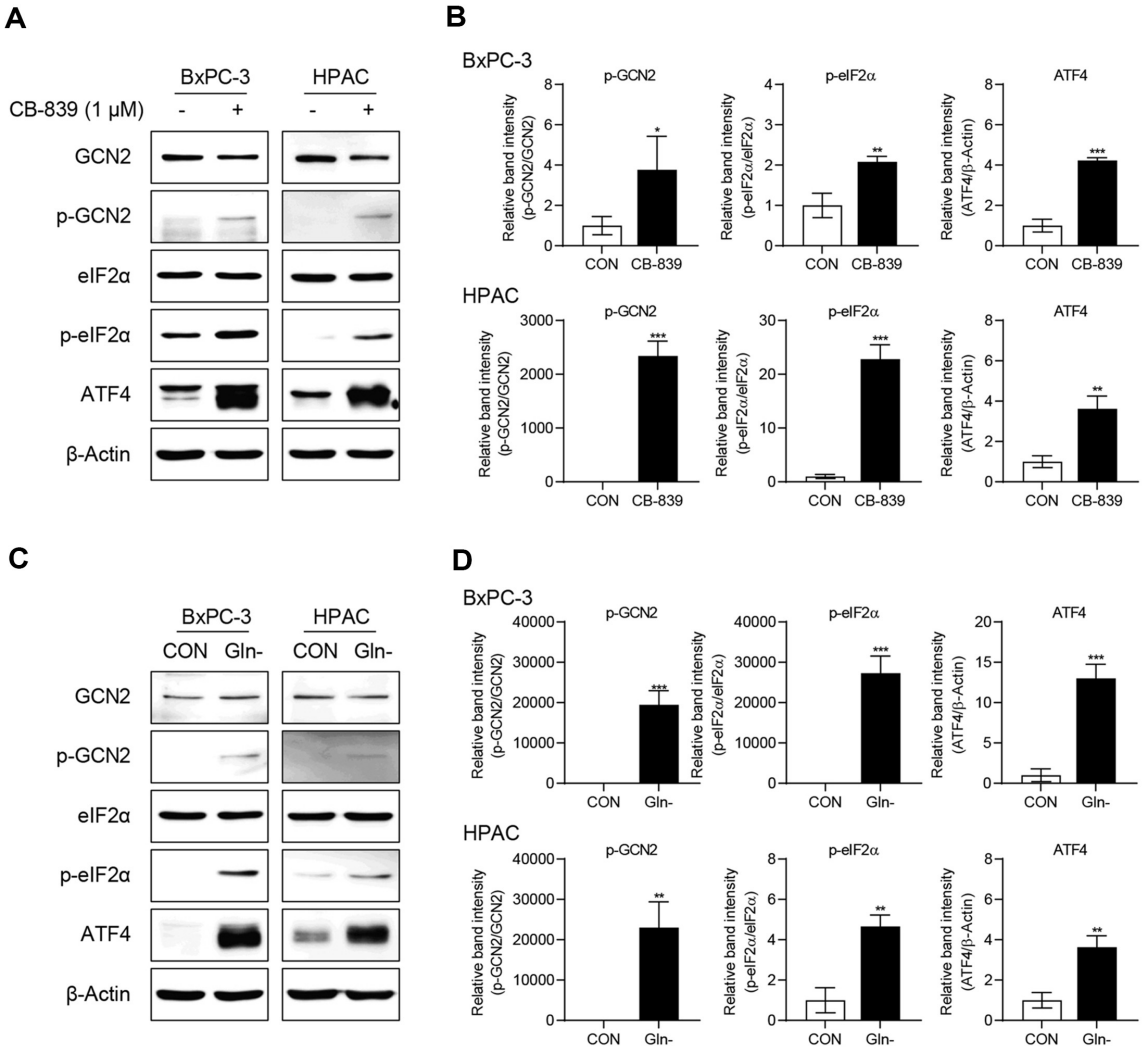
CB-839 treatment or glutamine deprivation increases ATF4 expression via the GCN2–ATF4 signaling pathway. (**A**, **B**) BxPC-3 and HPAC cells were seeded in 100-mm dishes and allowed to stabilize for 24 h. Each cell lines were then treated with 1 μM CB-839 for an additional 24 h. The band intensities of p-GCN2, p-eIF2α, and ATF4 were quantified using ImageJ and normalized to GCN2, eIF2α, and β-Actin, respectively, to determine relative expression levels. Statistical analyses were performed using GraphPad Prism 8 with a Student’s *t*-test. Error bars indicate the mean ± SD (*n* = 3). *p < 0.05, **p < 0.01, and ***p < 0.001 compared with the CON vs. CB-839 treated group. (**C**, **D**) BxPC-3 and HPAC cells were seeded in 100-mm dishes and allowed to stabilize for 24 h. Each cell lines were then cultured in glutamine-free medium for an additional 24 h. The band intensities of p-GCN2, p-eIF2α, and ATF4 were quantified using ImageJ and normalized to GCN2, eIF2α, and β-Actin, respectively, to determine relative expression levels. Statistical analyses were performed using GraphPad Prism 8 with a Student’s *t*-test. Error bars indicate the mean ± SD (*n* = 3). *p < 0.05, **p < 0.01, and ***p < 0.001 compared with the CON vs. Gln− group.

Because CB-839 and glutamine deprivation reduced cell viability, the underlying mechanisms were assessed by examining the amino acid stress signaling pathways. Glutamine deprivation activates GCN2, a primary sensor of amino acid deficiency. Upon activation, GCN2 phosphorylates eIF2α, thereby suppressing general protein synthesis while selectively enhancing ATF4 translation [22, 23]. Our results revealed that both CB-839 (Fig. 2A and 2B) and glutamine deprivation (Fig. 2C and 2D) activated the GCN2-eIF2α-ATF4 signaling pathway, thereby enhancing ATF4 expression. This indicated that the inhibition of glutamine metabolism induces a stress response through this pathway, highlighting the potential role of ATF4 in cellular adaptation to amino acid stress.

### Reducing ATF4 Expression Reduces Cell Viability

**Fig. 3:**
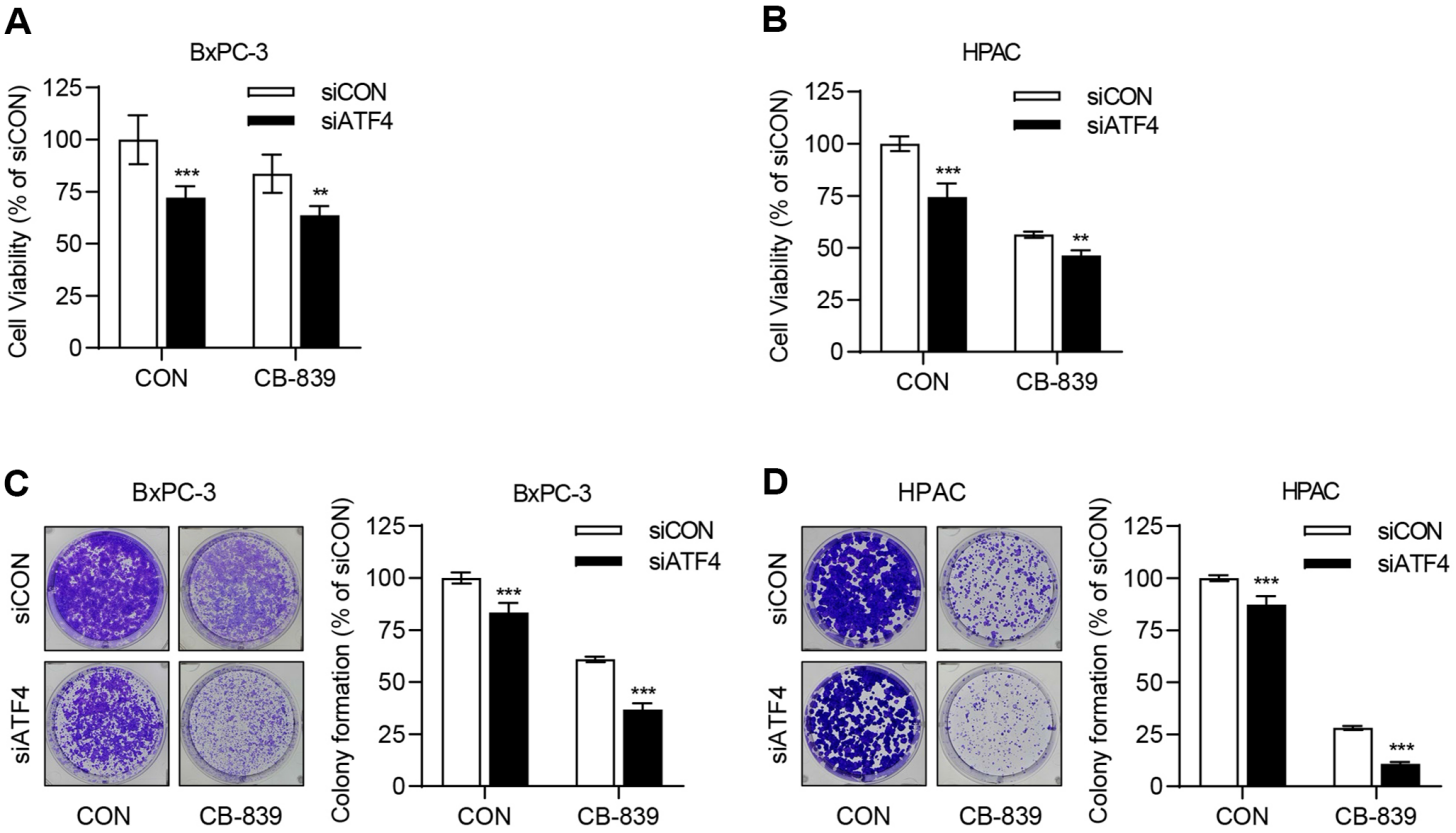
Effects of ATF4 knockdown on cell viability and colony formation. (**A**, **B**) BxPC-3 and HPAC cells were seeded into 96-well plates at 1.5 × 10^3^ and 1.0 × 10^3^ cells per well, respectively. One day later, the cells were transfected with siATF4 or siCON and allowed to stabilize. Subsequently, 1 μM CB-839 was added for 3 days, after which cell viability was evaluated using the MTT assay. Statistical significance was determined using two-way ANOVA followed by Tukey’s post hoc test in GraphPad Prism 8. Error bars represent the mean ± SD (*n* = 6). **p < 0.01 and ***p < 0.001 vs. CON. (**C**, **D**) To assess the impact of ATF4 knockdown on colony formation, BxPC-3 and HPAC cells were seeded into 6-well plates at 1.0 × 10^4^ and 1.0 × 10^3^ cells per well, respectively. After three days, transfection was performed using Oligofectamine reagent according to the established protocol with siCON or siATF4. The following day, cells were treated with 1 μM CB-839 or exposed to glutamine deprivation. Five days later, colonies were stained with crystal violet for quantification. Statistical analyses were performed using two-way ANOVA followed by Tukey’s test in GraphPad Prism 8. Error bars indicate the mean ± SD (*n* = 3). ***p < 0.001 vs. siRNA CON group.

To assess whether ATF4 facilitates cell survival or death, ATF4 was knocked down using siRNA under glutamine deprivation or in the presence of 1 μM CB-839. MTT assays revealed a marked reduction in cell viability in the ATF4 knockdown group compared to that in the control group (Fig. 3A and 3B). Similarly, colony formation assays demonstrated fewer colonies in ATF4-silenced cells under both conditions (Fig. 3C and 3D). These results indicated that ATF4 is crucial in facilitating cell survival during glutamine metabolic stress by potentially mitigating nutrient deprivation-induced cytotoxicity.

### Enhanced ATF4 Expression under Glutamine Deprivation Upregulates the Glutamine Transporter Expression

**Fig. 4:**
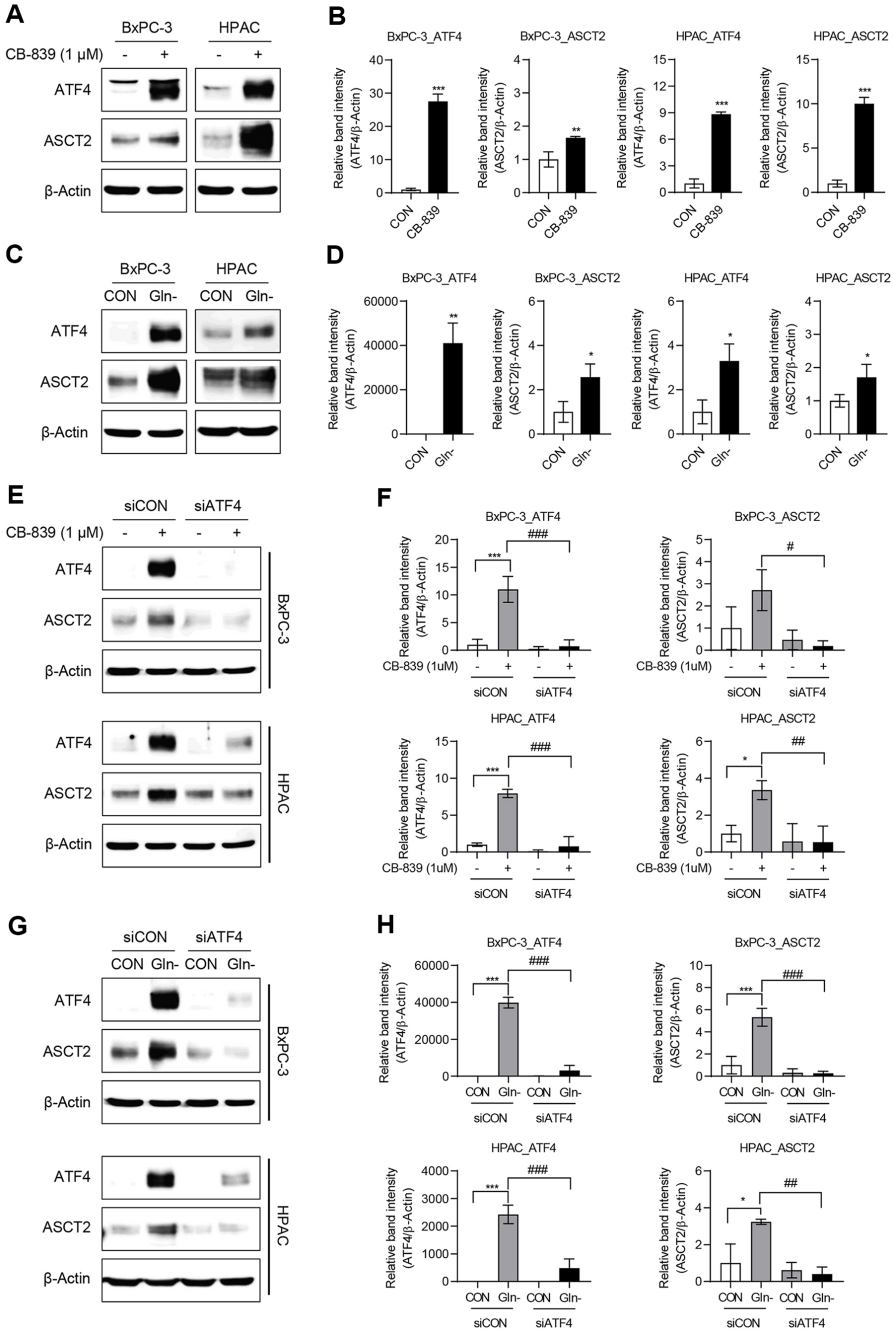
ATF4 upregulates ASCT2 expression under glutamine deprivation. (**A**–**D**) BxPC-3 and HPAC cells were seeded in 100-mm dishes and allowed to stabilize for 24 h. Each cell lines were then treated with 1 μM CB-839 or cultured in glutamine-free medium for an additional 24 h. The protein expression levels of ATF4 and ASCT2 were determined by Western blot analysis. Band intensities were measured using ImageJ and normalized to β-Actin. Statistical analyses were performed using Student’s *t*-test in GraphPad Prism 8. Error bars represent the mean ± SD (*n* = 3). *p < 0.05, **p < 0.01, and ***p < 0.001 compared with the CON group. (**E**–**H**) BxPC-3 and HPAC cells were transfected with either siCON or siATF4 using Oligofectamine according to the manufacturer’s protocol. After 24 h, cells were treated with 1 μM CB-839 or cultured in glutamine-free medium for an additional 24 h. Protein lysates were then collected, and Western blot analysis was performed to assess ATF4 and ASCT2 expression. β-Actin served as a loading control. Band intensities of ATF4 and ASCT2 were measured using ImageJ and normalized to β-Actin. Statistical analyses were conducted using two-way ANOVA followed by Tukey’s test in GraphPad Prism 8. Error bars indicate the mean ± SD (*n* = 3). *p < 0.05, **p < 0.01, and ***p < 0.001 compared with the siCONCON group; #*p* < 0.05, ##*p* < 0.01, and ###*p* < 0.001 compared with the siCON-CB839 or siCON-Gln- group.

Because glutamine deprivation and CB-839 treatment activate ATF4, its role in regulating the glutamine transporter ASCT2 was examined. Western blot analysis demonstrated enhanced ASCT2 expression under both CB-839 treatment (Fig. 4A and 4B) and glutamine deprivation (Fig. 4C and 4D). To assess whether ATF4 directly regulates ASCT2, an ATF4 knockdown experiment was conducted in same experiment conditions. The results revealed a substantial reduction in ASCT2 levels following ATF4 suppression under both CB-839 treatment (Fig. 4E and 4F) and glutamine deprivation (Fig. 4G and 4H), indicating that ATF4 transcriptionally regulates ASCT2 expression. This upregulation likely enhanced intracellular glutamine uptake, enabling cancer cells to adapt to metabolic stress and resist CB-839 treatment.

### ATF4 Facilitates Survival under Glutamine Metabolic Stress Conditions

**Fig. 5:**
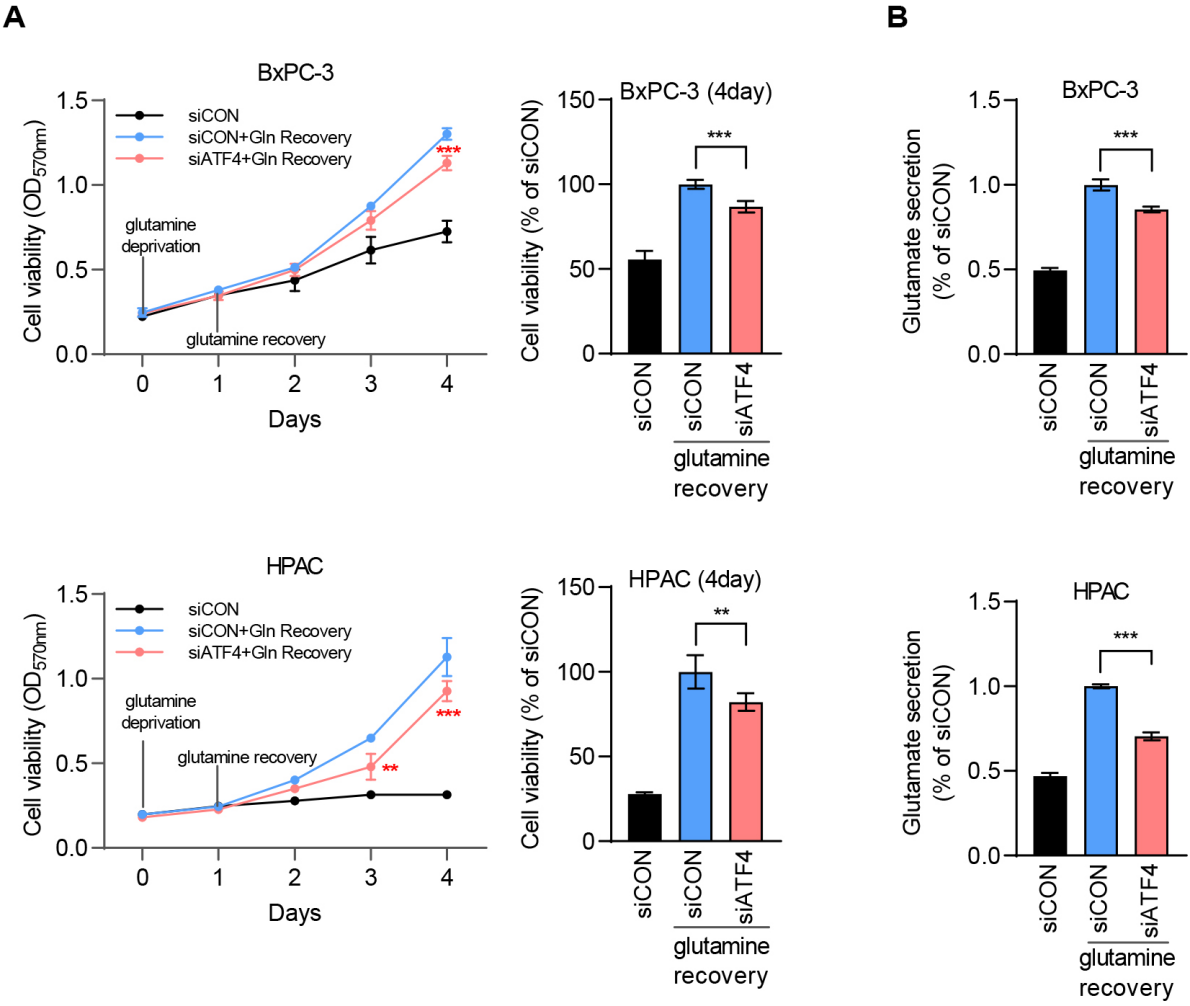
ATF4 facilitates cell survival under glutamine-deprivation. (**A**) To assess the effect of ATF4 on cell viability, cells were transfected with siATF4 or siCON. The cells were cultured under glutamine deprivation for one day to induce ATF4 expression, followed by either supplementation with 4 mM glutamine or continued glutamine deprivation. Subsequently, cell viability was measured using the MTT assay. Statistical analysis of 4 days samples was performed using GraphPad Prism 8, and differences in viability relative to the CON group were assessed using a two-way analysis of variance (ANOVA) followed by Tukey’s test. Error bars represent the mean ± standard deviation (SD) (*n* = 3). **p < 0.01 and ***p < 0.001 vs. siCON group. (**B**) To determine the level of glutamate secretion in the 4-day samples from each group, culture media were collected from each sample. Glutamate secretion was measured using the Glutamine/Glutamate-Glo Assay kit and normalized to the corresponding MTT assay data. Statistical significance was determined by two-way ANOVA followed by Tukey’s test in GraphPad Prism 8. Error bars represent the mean ± SD (*n* = 3). ***p < 0.001 vs. siCON group.

Because ATF4 enhances ASCT2 expression, its role in facilitating cell survival during glutamine stress was further assessed. Cells with and without ATF4 knockdown were subjected to a 1-day glutamine deprivation, followed by glutamine recovery. MTT assays revealed a substantial reduction in cell viability in the ATF4 knockdown group than that in the control group in both cell lines, indicating impaired recovery from glutamine deprivation (Fig. 5A). Additionally, glutamate levels, a primary metabolite of glutamine metabolism, were measured on the fourth day of recovery. The ATF4 knockdown group exhibited lower glutamate levels than that of the control group, indicating reduced glutamine utilization (Fig. 5B). These findings indicated that ATF4 enhances glutamine metabolism by upregulating ASCT2, thereby facilitating cell survival under glutamine-limited conditions.

### Combination Treatment of V-9302 and CB-839 Effectively Reduces Cell Viability

**Fig. 6:**
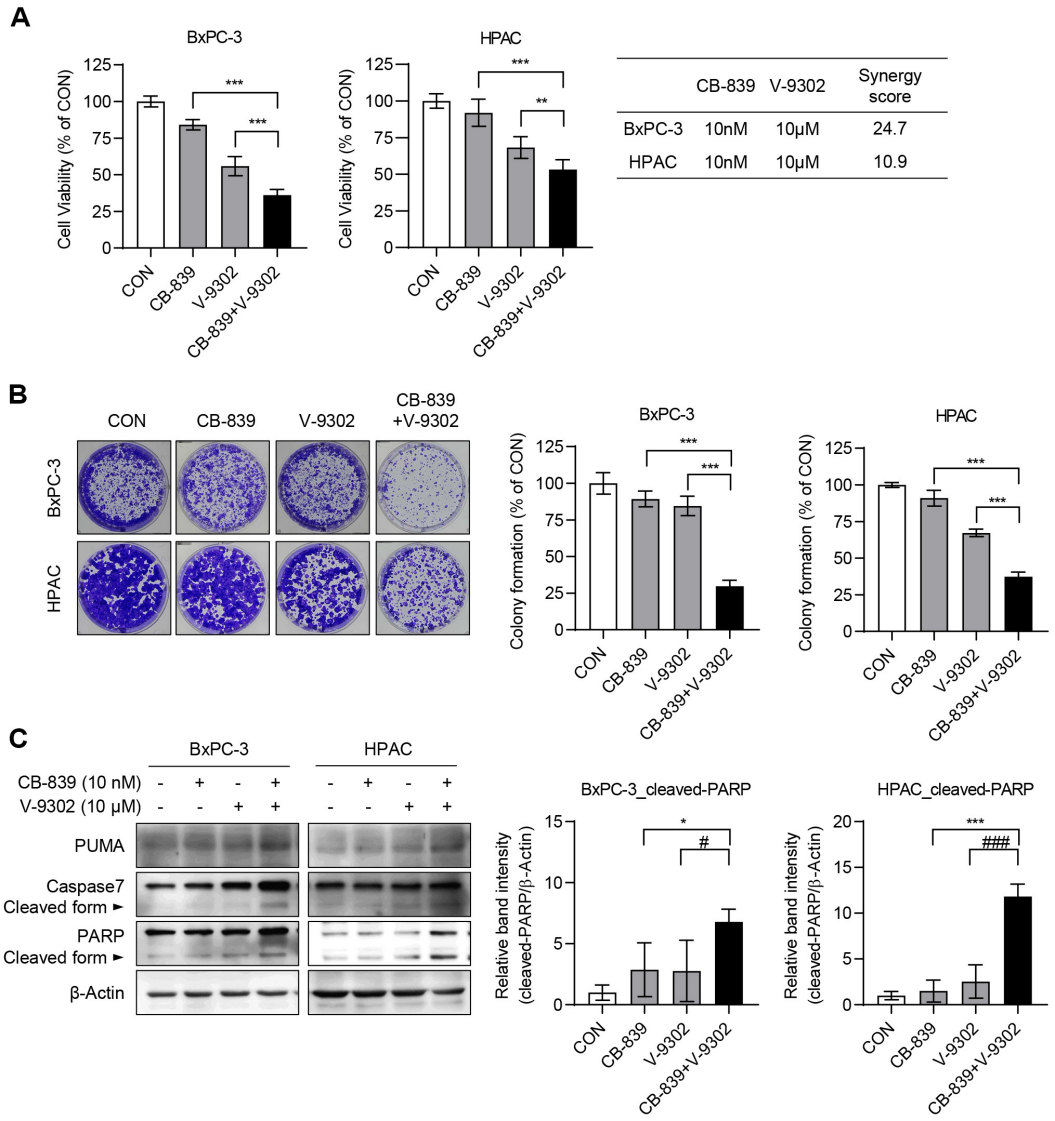
Evaluation of cell viability and apoptosis following combined treatment with V-9302 and CB-839. (**A**) To assess the combined effects of V-9302 and CB-839 on cell viability, MTT assay was performed. Cells were treated with 10 μM V-9302 or 10 nM CB-839, both individually and in combination, for 3 days, after which cell viability was quantified using the MTT assay. Statistical analyses were conducted using GraphPad Prism 8; differences in cell viability relative to the CON group were determined by one-way ANOVA followed by Tukey’s post hoc test. Data are presented as the mean ± standard deviation (SD) (*n* = 6), with **p < 0.01 and ***p < 0.001 between the indicated group. The synergistic effect of the CB-839 + V-9302 group in BxPC-3 and HPAC was confirmed using the SynergyFinder web-tool. A synergy score of ≥10 is considered to be strong synergistic effect. (**B**) For the colony formation assay, BxPC-3 and HPAC cells were seeded in 6-well plates at densities of 1 × 10^4^ and 1 × 10^3^ cells per well, respectively, and incubated for 4 days. Thereafter, cells were treated with 10 μM V-9302 or 10 nM CB- 839, both individually and in combination, for 5 days. Colonies were fixed with methanol and stained with crystal violet for quantification. Statistical analysis was performed by one-way ANOVA followed by Tukey’s post hoc test. Data are expressed as the mean ± SD (*n* = 3), with ***p < 0.001 between the indicated group. (**C**) To evaluate the expression of apoptotic marker proteins, cells were treated with CB-839 (10 nM) or V-9301 (10 μM), both individually and in combination, for 3 days. Proteins were extracted and subjected to Western blot analysis to assess the expression levels of apoptosis-related proteins, with β-Actin serving as the loading control. Band intensities of cleaved-PARP were measured using ImageJ and normalized to β-Actin. Statistical analyses were conducted using one-way ANOVA followed by Tukey’s test in GraphPad Prism 8. Error bars indicate the mean ± SD (*n* = 4). **p < 0.01, and ***p < 0.001 compared with the CB-839 group; ##*p* < 0.01, and ###*p* < 0.001 compared with the V-9302 group.

Because ATF4-induced glutamine transporters, such as ASCT2 confer resistance to CB-839, we hypothesized that inhibiting these transporters might enhance cancer cell sensitivity to CB-839. To test this, cells were treated with a low concentration of CB-839 (10 nM) and V-9302 (10 μM), either alone or in combination. MTT assays revealed a significant reduction in cell viability following combination treatment compared to treatment with either agent alone (Fig. 6A). Additionally, we analyzed the synergistic effect of the CB-839+V-9302 group in BxPC-3 and HPAC cell lines using the SynergyFinder web-tool. As a result, the respective synergy scores were determined to be 24.7 and 10.9. In general, if the synergy score is higher than 10, it is considered indicative of a strong synergistic interaction. Therefore, the combined treatment of CB-839 and V-9302 was confirmed to be highly effective in reducing cell viability. Similarly, colony formation assays also revealed a significant reduction in colony-forming ability with combination treatment (Fig. 6B). These results indicated that co-targeting glutamine metabolism and transport exerts a synergistic effect, effectively reducing cancer cell viability.

### Combination Treatment of CB-839 and V-9302 Induces Apoptosis

To assess whether the observed reduction in cell viability was because of apoptosis, apoptotic markers were examined using western blot analysis. The results revealed increased levels of cleaved caspase-3 and PARP in cells treated with the CB-839 and V-9302 combination, indicating enhanced apoptosis induction (Fig. 6C). This indicates that the combination treatment effectively induced apoptotic cell death, contributing to the reduction in pancreatic cancer cell viability.

## Discussion

Cancer cells depend on glutamine transporters to meet the increased demand for glutamine that is essential for rapid proliferation, biosynthesis, and redox balance. Various primary transporters, including B0AT1(SLC6A19), LAT1 (SLC7A5), SNAT5 (SLC38A5), and ASCT2 (SLC1A5) [24], play significant roles in glutamine uptake by cancer cells. ASCT2 is crucial because it is overexpressed in various cancers and correlates with increased glutamine dependence, making it a significant target for therapeutic intervention [16, 21].

Cancer cells are limited by metabolic flexibility—where they use other nutrients when one nutrient is scarce, and metabolic plasticity—where they alter pathways [12]. For example, in arginine-dependent hepatocellular carcinoma, limiting arginine supply inhibits growth. Hepatocytes respond to arginine deficiency by upregulating the arginine transporter SLC7A1 expression through enhanced GCN2 expression [25]. Therefore, the use of metabolic inhibitors in cancer therapy may be limited by the complexity of metabolic pathways and bypasses. Our findings demonstrate that ASCT2 expression is upregulated by the ATF4 signaling pathway in response to cellular stress signals, such as glutamine deprivation or GLS inhibition. This adaptive mechanism enables cancer cells to increase glutamine uptake and sustain metabolic demands, despite external stress. Consequently, targeting ASCT2-mediated glutamine transport may reduce glutamine availability and sensitize cancer cells to glutamine metabolism inhibitors, such as CB-839 [16, 26].

The metabolic plasticity of cancer cells complicates therapeutic targeting because they adapt to nutrient deprivation by activating alternative pathways, such as ISR. The ISR signaling pathway is crucial for cell survival and adaptation to stimuli, including amino acid deficiency, oxidative stress, and mitochondrial stress [27-29]. ISR involves eIF2α phosphorylation, which inhibits global protein synthesis while selectively enhancing the translation of stress-responsive proteins, such as ATF4. Four kinases—GCN2, protein kinase RNA-like endoplasmic reticulum kinase (PERK), protein kinase R (PKR), and heme-regulated inhibitor (HRI)—regulate ISR activation through eIF2α phosphorylation. GCN2 is activated through amino acid deprivation by sensing uncharged tRNA, whereas PERK responds to endoplasmic reticulum stress. PKR is activated by viral infections through double-stranded RNA recognition, whereas HRI is triggered by heme deficiency or oxidative stress [27, 29]. GCN2 plays a crucial role in responding to amino acid deprivation by sensing uncharged tRNA and inducing ATF4 translation. When glutamine is deficient, GCN2 is activated by recognizing uncharged tRNA [22, 23]. Activated GCN2 (eIF2α kinase) induces eIF2α phosphorylation, which inhibits the formation of the eIF2α ternary complex required for translation, thereby reducing the translation of most messenger RNAs (mRNAs) [30, 31]. In contrast, mammalian ATF4 mRNA has an inhibitory Open Reading Frame upstream that binds to the eIF2α ternary complex, resulting in enhanced translation owing to eIF2α phosphorylation. Therefore, eIF2α phosphorylation inhibits translation by reducing the ternary complex, but it specifically enhances ATF4 translation [29, 32, 33].

ATF4 acts as a transcription factor that regulates the genes associated with amino acid transport, oxidative stress protection, and protein homeostasis. Although ATF4 can facilitate apoptosis in certain contexts [34-37], its primary role in cancer cells is to facilitate survival and adaptation [38, 39]. This dual functionality indicated that ATF4 can drive resistance mechanisms in response to glutamine metabolism inhibitor-induced metabolic stress.

Our experiments confirmed that CB-839 treatment induces ATF4 expression in pancreatic cancer cells through the GCN2-ATF4 signaling pathway. ATF4 knockdown reduced cell viability under glutamine-deprived conditions, indicating that ATF4-mediated stress responses supported cancer cell survival. Additionally, ATF4 enhanced ASCT2 expression, facilitating glutamine uptake and conferring resistance to CB-839. These findings aligned with previous reports, indicating that ATF4 played a crucial role in enabling cells to adapt to nutritional stress by upregulating amino acid transporters and metabolic enzymes essential for survival under nutrient-limited conditions [14, 17, 29, 40]. This adaptive response may explain why CB-839 monotherapy is less effective in certain situations, owing to the activation of compensatory mechanisms that restore intracellular glutamine levels through enhanced transporter activity. Therefore, these findings support the hypothesis that dual inhibition of ASCT2 and GLS is more effective than targeting GLS alone [41].

To test this hypothesis, we treated pancreatic cancer cells with low concentrations of CB-839 and V-9302. MTT and colony formation assays revealed a significant reduction in cell viability and colony formation when both agents were combined compared to either treatment alone. This synergy highlights the therapeutic potential of targeting glutamine transport alongside GLS inhibition. However, despite the promising results, it is important to acknowledge the potential for long-term resistance development to this combination therapy. Cancer cells exhibit metabolic plasticity, which allows them to adapt to nutrient deprivation by activating alternative metabolic pathways or compensatory mechanisms. For example, cancer cells may upregulate other amino acid transporters or rely on alternative nutrient sources such as glucose or fatty acids to sustain their metabolic demands [42, 43] Future studies should focus on investigating these potential resistance mechanisms and identifying strategies to overcome them. This could include exploring additional combination therapies that target compensatory pathways, such as inhibitors of glycolysis, fatty acid metabolism, or autophagy.

In addition to ISR signaling pathways such as GCN2-ATF4 activation under glutamine deprivation conditions, cancer cells can activate alternative metabolic pathways to compensate for nutrient stress. For instance, mammalian target of rapamycin complex 1 (mTORC1) activation promotes glutaminolysis and cell growth while repressing autophagy. Similarly, AMP-activated protein kinase (AMPK) activation under nutrient stress suppresses mTORC1 activity and supports energy homeostasis through alternative energy production mechanisms [44]. Moreover, GLS1 inhibition has been shown to induce pyruvate carboxylase-mediated anaplerosis to replenish tricarboxylic acid (TCA) cycle intermediates independently of glutamine [43]. These adaptive mechanisms highlight the need for combination therapies targeting multiple pathways to overcome resistance.

CB-839 has been evaluated in clinical studies across various cancer types, including RCC, where it demonstrated promising results when combined with agents such as cabozantinib or everolimus. These findings highlight the potential of CB-839 as part of combination therapies targeting metabolic cancer [45, 46]. However, in pancreatic cancer cells, our findings indicate the presence of metabolic adaptive mechanisms, such as ATF4-mediated upregulation of ASCT2 expression. These mechanisms suggest that CB-839 monotherapy may exhibit limited efficacy in clinical trials for pancreatic cancer. Indeed, other preclinical studies have reported that while GLS inhibitors show potent efficacy in *in vitro* models, these effects are not consistently replicated *in vivo* [47]. This discrepancy can be attributed to the metabolic flexibility of pancreatic cancer cells, which adapt to glutamine metabolism inhibition by activating alternative pathways or utilizing other nutrients, such as glucose or fatty acids. Therefore, the successful clinical application of CB-839 in pancreatic cancer will likely require combination therapies targeting metabolic adaptive pathways. Such multi-pathway targeting strategies could overcome metabolic resistance in pancreatic cancer and maximize the therapeutic efficacy of CB-839.

**Fig. 7:**
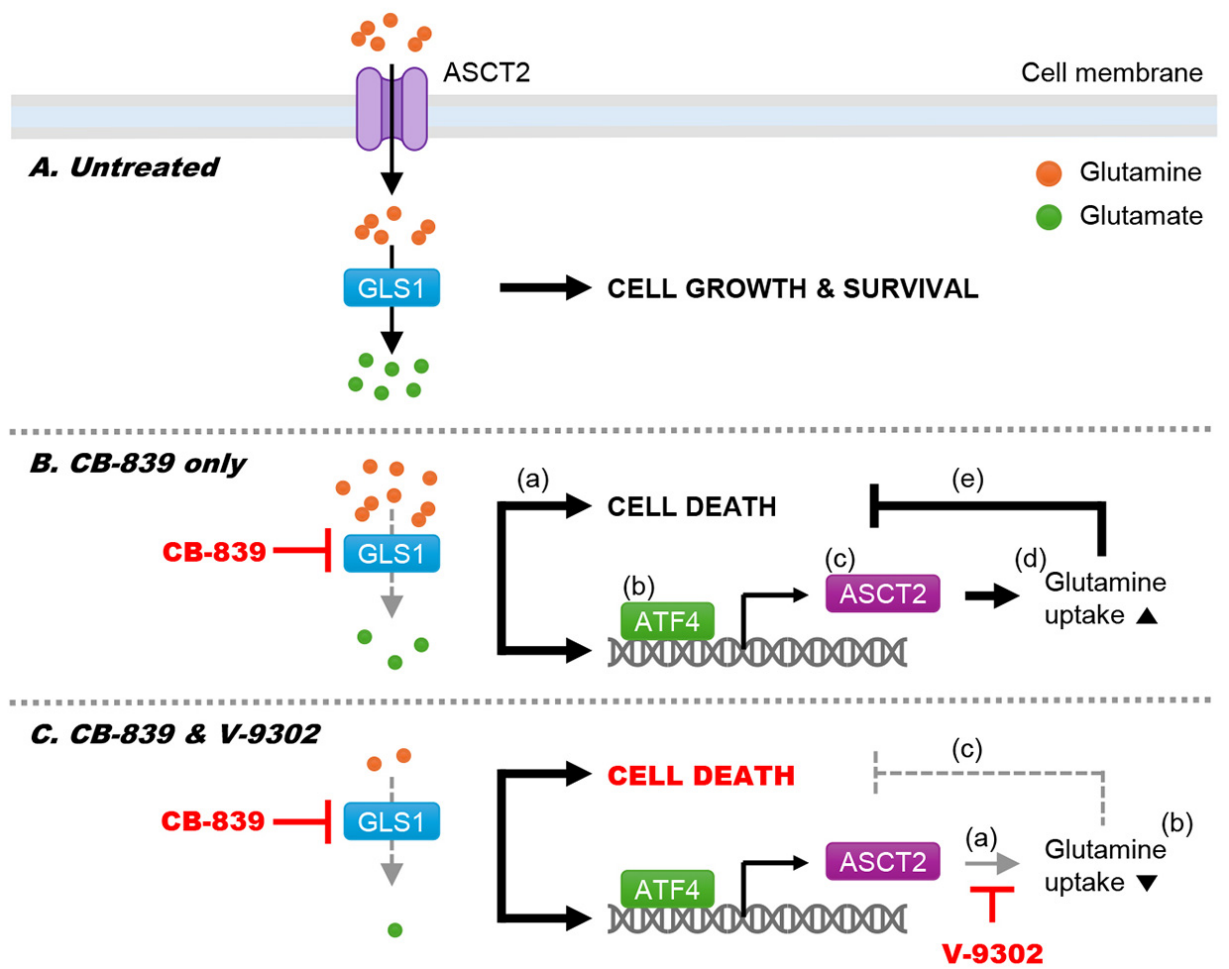
Schematic diagram of proposed model. (**A**) Under untreated conditions, glutamine enters the cell via ASCT2 and is converted to glutamate by GLS1, thereby promoting cancer cell growth and survival. (**B**) When treated with CB-839 alone, GLS1 activity is inhibited, leading to reduced glutamate production and the induction of metabolic stress, which triggers cell death (a). However, the decline in glutamate also activates ATF4 (b), resulting in increased ASCT2 expression (c) and enhanced glutamine uptake (d), partially counteracting the cell death effect (e). (**C**) Under combined treatment with CB-839 and V-9302, the upregulated ASCT2 expression induced by glutamate depletion is effectively suppressed (a), preventing excessive glutamine influx (b). Consequently, the metabolic stress caused by glutamate depletion is exacerbated, ultimately amplifying cancer cell death (c).

In summary, our findings demonstrate that ATF4-mediated upregulation of ASCT2 contributes to resistance against CB-839 in pancreatic cancer cells (Fig. 7). Combining CB-839 with V-9302 effectively mitigates this adaptive response and enhances anticancer efficacy. However, our study has several limitations that should be addressed. First, while we demonstrated the synergistic effect of dual inhibition of ASCT2 and GLS1 using CB-839 and V-9302, the potential off-target effects and toxicity of this combination therapy were not thoroughly investigated. Second, our study primarily focused on the GCN2-ATF4 signaling pathway, but other compensatory pathways or mechanisms that may contribute to metabolic plasticity and therapeutic resistance were not explored. Additionally, our findings are based on in vitro experiments, and further in vivo validation is necessary to assess the translational potential of this strategy. Future studies should evaluate this combination therapy in other tumor types and investigate its efficacy in conjunction with additional treatments, such as immune checkpoint inhibitors or chemotherapeutic agents, to enhance clinical outcomes.
